# Puerarin inhibits FUNDC1-mediated mitochondrial autophagy and CSE-induced apoptosis of human bronchial epithelial cells by activating the PI3K/AKT/mTOR signaling pathway

**DOI:** 10.18632/aging.203317

**Published:** 2022-02-08

**Authors:** Li Wang, Weizhou Jiang, Jing Wang, Yuanyuan Xie, Weisi Wang

**Affiliations:** 1 Department of Respiratory Medicine, Yan’an University Affiliated Hospital, Yan’an 716000, China; 2 Department of Pulmonology, Weifang Traditional Chinese Hospital, Weifang 261041, China; 3 Endoscopy Room, Tai’an Maternal and Child Health Hospital, Tai’an 271000, China; 4 Department of Geriatrics, Yan’an University Affiliated Hospital, Yan’an 716000, China; 5 Department of Respiratory Medicine, The Second Affiliated Hospital of Zhejiang Chinese Medical University, Hangzhou 310005, China

**Keywords:** chronic obstructive pulmonary disease, puerarin, mitochondrial autophagy, FUNDC1, PI3K/AKT/mTOR signaling pathway

## Abstract

Increasing evidence suggests that the pathogenesis of chronic obstructive pulmonary disease (COPD) is associated with FUN14 domain protein 1 (FUNDC1)-mediated mitophagy. Recently, studies have reported that puerarin has protective effects against excessive oxidative damage in cells. Therefore, we hypothesized that puerarin may be involved in COPD progression via regulating FUNDC1 mediated mitophagy. We found that the viability of cigarette smoke extract (CSE)-stimulated human bronchial epithelial cells (HBECs) was enhanced and apoptosis was reduced after treatment with different concentrations of puerarin. Puerarin reversed mitochondrial membrane potential (MMP) levels and ATP content, and decreased reactive oxygen species (ROS) content in CSE stimulated HBECs. Moreover, puerarin significantly inhibited apoptosis related proteins, as well as the expression of mitophagy related proteins. After inhibition of FUNDC1 phosphorylation by protein phosphatase inhibitor (PH0321), puerarin restored MMP level, decreased ROS content, promoted ATP synthesis, and downregulated autophagy related protein expression in HBECs. In addition, mitochondrial division inhibitor (Mdivi) inhibited the expression of autophagy related proteins and reduced apoptosis after blocking cell autophagy, which was the same as the inhibition of puerarin. Finally, puerarin activated the PI3K/Akt/mTOR signaling pathway to participate in COPD progression by up regulating the phosphorylation levels of PI3K, Akt and mTOR.

## INTRODUCTION

Chronic obstructive pulmonary disease (COPD) is a common disease characterized by persistent airflow limitation, and its clinical manifestations are mainly chronic cough, shortness of breath, chest tightness or wheezing [1, 2]. Persistent chronic inflammation induces the recurrence of tracheal wall injury and repair process, leading to airway remodeling, which is the main cause of irreversible progression of COPD [3, 4]. The morbidity and mortality of COPD are increasing year by year, which seriously affects the working ability and quality of life of patients and causes a heavy socio-economic burden.

Smoking is the most important risk factor for COPD [5]. Cigarette smoke constantly stimulates the alveoli, leading to the elevation of endogenous reactive oxygen species (ROS) in inflammatory cells and epithelial cells, and mitochondrial damage, which in turn induces the imbalance between oxidative and antioxidant processes in cells [6]. It has been shown that cigarette smoke extract (CSE)-stimulated human bronchial epithelial cells (HBECs) exhibit stronger mitophagy [7, 8]. In COPD progression, mammalian mitotic receptor FUN14 domain protein 1 (FUNDC1) is closely related to autophagy and apoptosis in hypoxic cells, which can participate in mitosis through enhanced mitophagy [9]. Wen et al. found that silencing the FUNDC1 gene in CSE-stimulated HBECs, the expression of DRP1 was also inhibited, thus inhibiting mitophagy and apoptosis and slowing down the COPD process [10].

At present, most of the acute treatment options for COPD are antibiotics, bronchodilators, glucocorticoids and expectorants, but these treatment measures could not effectively delay or prevent the recurrence and progressive progress of the disease. Studies have shown that flavonoids have anti-inflammatory and antioxidant properties, which can effectively improve lung function and reduce inflammatory cell infiltration in chronic airway inflammation [11–13]. Gao et al. showed that Icariside II could protect PC12 cells from H_2_O_2_-induced death by inhibiting mitochondrial-mediated autophagy [14].

Puerarin is an isoflavone compound isolated from the dried root of *Pueraria lobata*, a traditional Chinese medicine. Its medicinal value is benefited from its wide range of pharmacological properties, such as vasodilation, cardio-protection, antioxidant, anti-apoptotic and reducing insulin resistance [15]. Puerarin plays a protective role against cell damage caused by pathological factors [16]. Puerarin has been reported to reduce Caspase-3 expression by activating PI3K/AKT signaling pathway, and significantly improve tumor necrosis factor-α (TNF-α) induced apoptosis in PC12 cells [17]. In addition, Chen et al. showed that puerarin can directly act on myocytes, alleviate mitochondrial dysfunction, mitosis and inflammatory response induced by palmitate, thereby contributing to the improvement of insulin sensitivity and the improvement of impaired insulin signaling in skeletal muscle and isolated myotubes of diabetic animals [18]. As mentioned above, puerarin may play an active role in targeted therapy of chronic obstructive pneumonia disease.

## RESULTS

### Puerarin promotes the proliferation and inhibits apoptosis of CSE-induced HBECs

**Figure 1:**
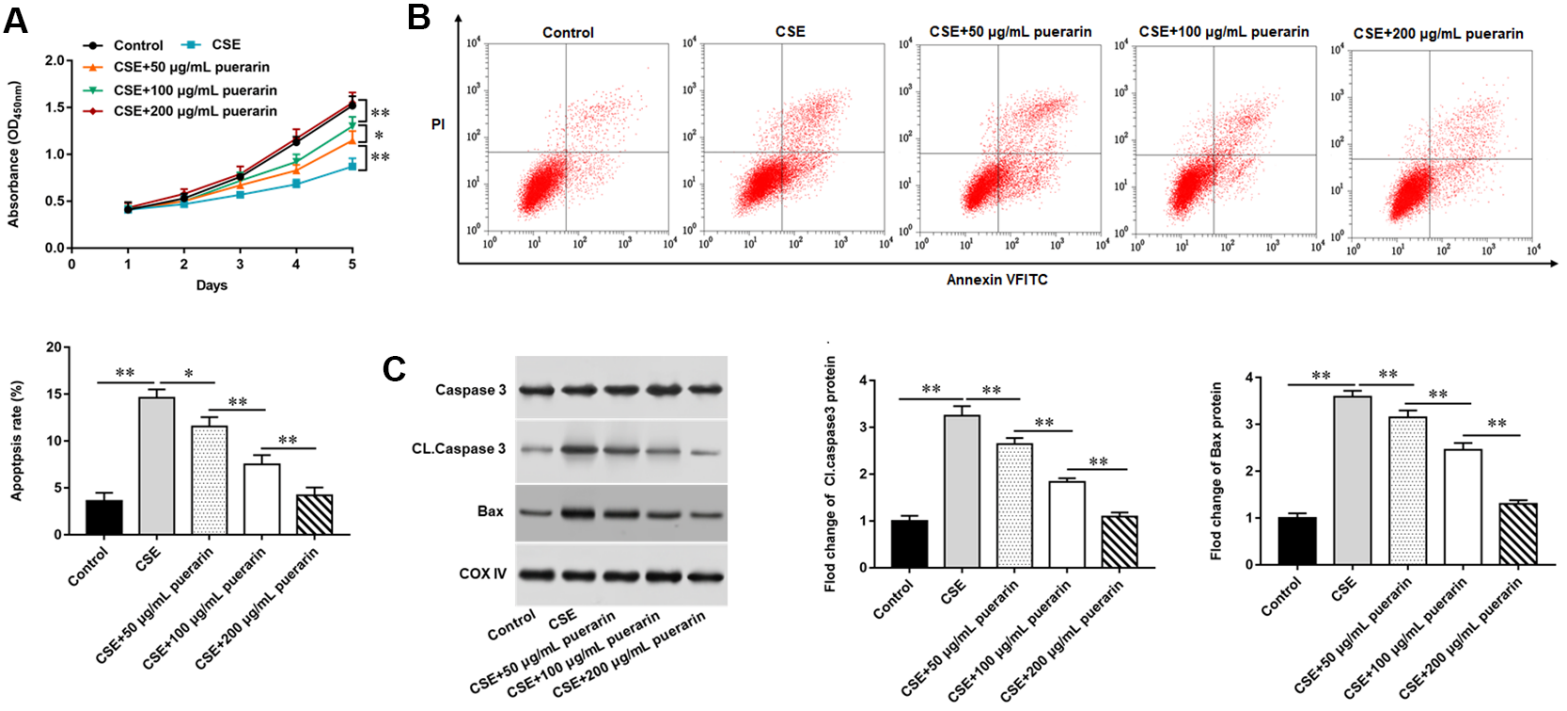
**Puerarin promotes the proliferation and inhibits apoptosis of CSE-induced HBECs.** HBECs was induced by 20% CSE and intervened with 50 μg/mL, 100 μg/mL and 200 μg/mL puerarin for 12 h, respectively. (**A**) MTT assay was used to measure the viability of CSE-induced HBECs. (**B**) Apoptosis of CSE-induced HBECs were detected by flow cytometry. (**C**) The protein expression of Cleaved caspase3 and Bax in CSE-induced HBECs were analyzed by Western blotting. β-actin was used as an invariant internal control for calculating protein-fold changes. N=6, * *P*<0.05, ** *P*<0.01.

To further explore the effect of puerarin on the proliferation and apoptosis of CSE-induced HBECs, different concentrations of puerarin were used to intervene in model cells. MTT assay results showed that the gradual increase of puerarin concentration could significantly promote the proliferation of HBECs compared with 20% CSE treatment alone (Figure 1A). Next, the results of flow cytometry showed that the apoptosis of HBECs treated with 20% CSE was significantly increased, but this trend was reversed after puerarin intervention at different concentrations, and the inhibition of apoptosis by high concentrations of puerarin was more significant (Figure 1B). Western blotting results suggested that the expression of apoptosis-related genes, including Cleaved caspase3 and Bax, decreased after puerarin intervention and was significantly correlated with the concentration (Figure 1C).

### Puerarin inhibits mitochondrial autophagy of CSE-induced HBECs

**Figure 2:**
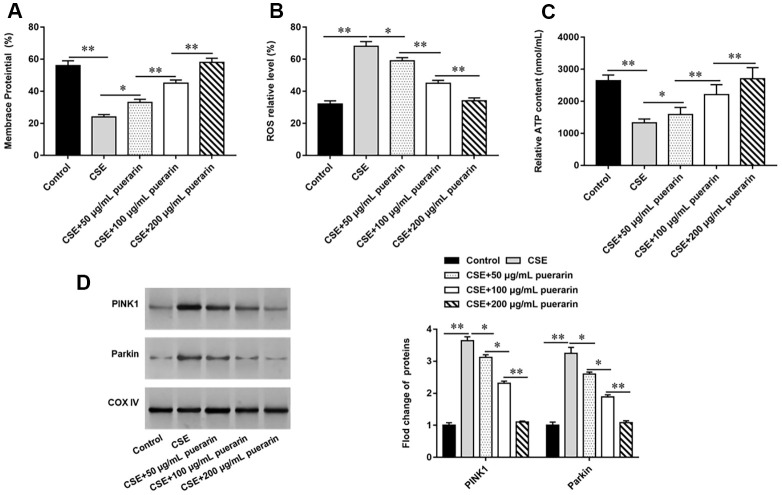
**Puerarin inhibits mitochondrial autophagy of CSE-induced HBECs.** HBECs was induced by 20% CSE and intervened with 50 μg/mL, 100 μg/mL and 200 μg/mL puerarin for 12 h, respectively. (**A**) Flow cytometry (JC-1) was used to detect changes in MMP level in CSE-induced HBECs. (**B**) Mitochondrial ROS levels were detected by flow cytometry (DCFH-DA). (**C**) The content of ATP in CSE-induced HBECs was detected with kits. (**D**) Western blotting was used to detect the expression of mitochondrial autophagy-related proteins such as PINK1 and Parkin. β-actin was used as the loading control. N=6, * *P*<0.05, ** *P*<0.01.

To clarify the effect of puerarin on mitochondria, puerarin with concentrations of 50 μg/ml, 100 μg/ml and 200 μg/ml was used to interfere with 20% CSE-induced HBECs, respectively. The changes of MMP level were detected by flow cytometry after labeling with JC-1 probe and the results showed that MMP level was significantly decreased after 20% CSE treatment, suggesting early apoptosis of cells. However, the decrease of membrane potential was gradually reversed after puerarin intervention at different concentrations, and was significantly correlated with puerarin concentration (Figure 2A). In addition, the results of flow cytometry suggested that ROS levels in HBECs increased significantly after 20% CSE treatment, while puerarin intervention could down-regulate ROS levels in cells, which was significantly correlated with the concentration (Figure 2B). Mitochondria are the main site of ATP synthesis. Treating cells with 20% CSE leads to damage of mitochondrial structure and function, and ATP content in cells decreases. Puerarin at different concentrations can significantly improve mitochondrial damage and increase the amount of ATP synthesis (Figure 2C). Western blotting was used to detect the expression levels of mitochondrial autophagy-related proteins. The results showed that the protein expression of PINK1 and Parkin decreased significantly with the increase of puerarin intervention concentration compared with 20% CSE treatment alone, suggesting that puerarin had an inhibitory effect on mitochondrial autophagy (Figure 2D).

### Puerarin down-regulates the expression of DRP1 and FUNDC1

**Figure 3:**
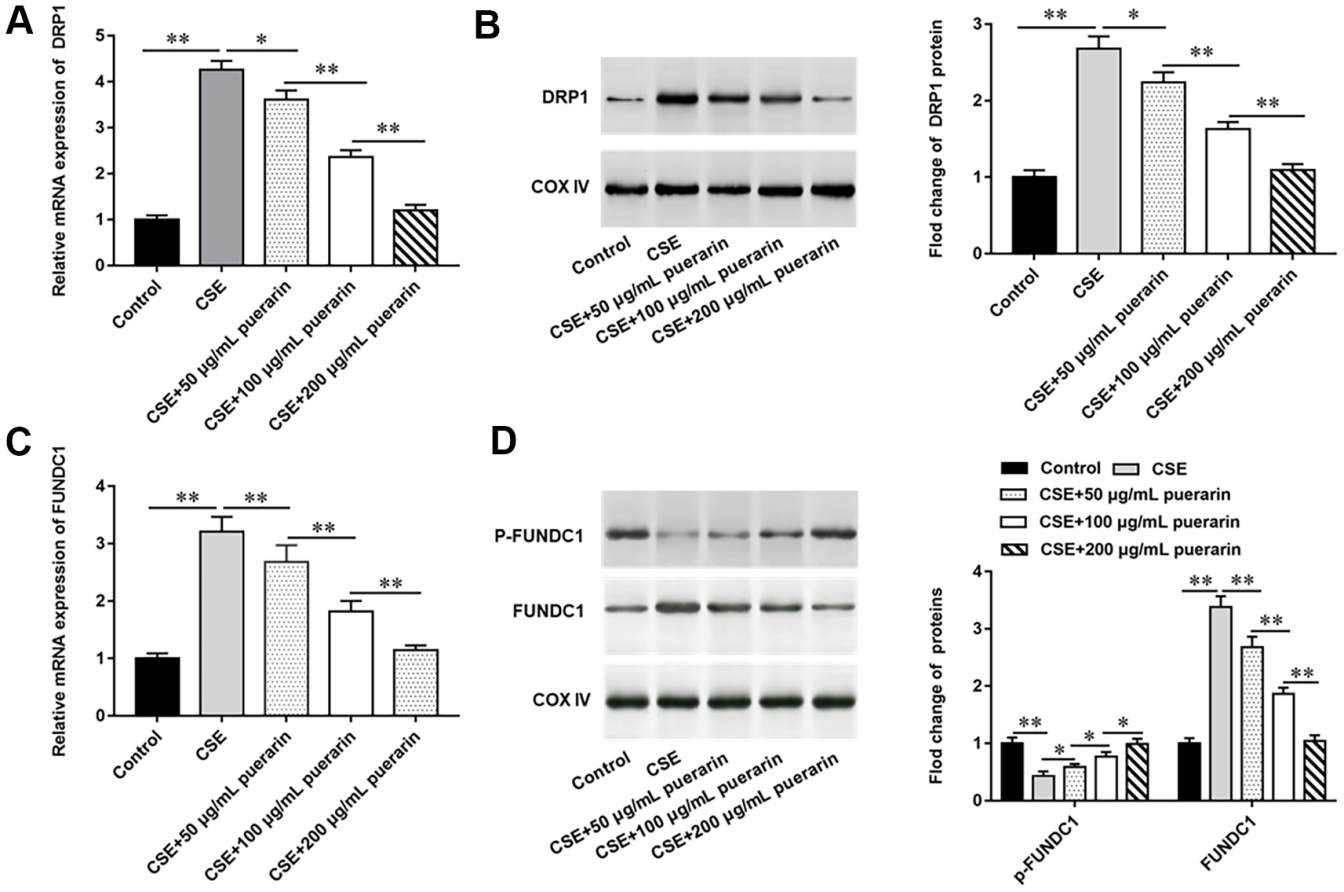
**Puerarin down-regulates the expression of DRP1 and FUNDC1.** HBECs was induced by 20% CSE and intervened with 50 μg/mL, 100 μg/mL and 200 μg/mL puerarin for 12 h, respectively. (**A**) Relative mRNA expression of DRP1 was analyzed by RT-qPCR. (**B**) Western blotting was used to measure the protein expression of DRP1. (**C**) Relative expression of FUNDC1 was detected by RT-qPCR. (**D**) The protein expression of FUNDC1 and p-FUNDC1 in CSE-induced HBECs were analyzed by Western blotting. β-actin was used as an internal reference. N=6, * *P*<0.05, ** *P*<0.01.

To investigate whether CSE-induced autophagy is associated with DRP1 and FUNDC1, the expression of DRP1 and FUNDC1 was measured. The results of RT-qPCR and Western blotting showed that DRP1 mRNA and protein expression were significantly increased after treatment of cells with 20% CSE, but this trend was reversed with the increasing concentration of puerarin intervention (Figure 3A, 3B). Similarly, the expression of FUNDC1 mRNA and protein was significantly up-regulated in 20% CSE-treated group, but its expression was down-regulated after puerarin intervention and significantly correlated with the concentration (Figure 3C, 3D). In addition, Western blotting results showed that expression of phosphorylated FUNDC1 protein was significantly down-regulated after 20% CSE treatment, and puerarin intervention at different concentrations could significantly reverse its expression, which was correlated with the concentration. This suggests that FUNDC1 inhibits mitochondrial autophagy in the form of phosphorylation (Figure 3D).

### Puerarin prevents mitochondrial autophagy by inhibiting dephosphorylation of FUNDC1

**Figure 4:**
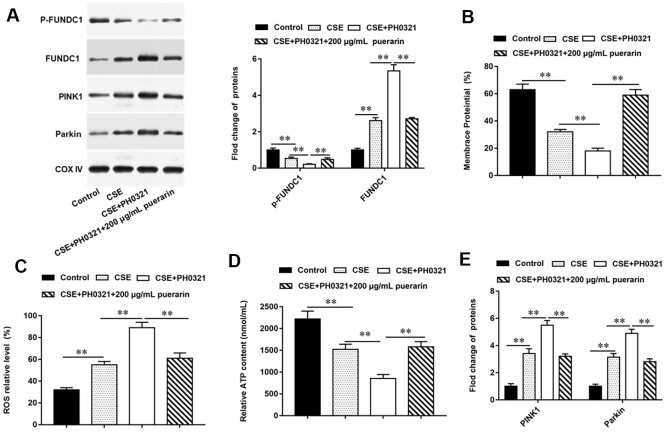
**Puerarin prevents mitochondrial autophagy by inhibiting dephosphorylation of FUNDC1.** PH0321, a protein phosphatase inhibitor, treated 20% CSE-induced HBECs and then co-cultured with puerarin at the concentration of 200 μg/mL. (**A**) The protein expression of FUNDC1 and p-FUNDC1 in CSE-induced HBECs were analyzed by Western blotting. (**B**) Flow cytometry (JC-1) was used to detect changes in mitochondrial membrane potential in CSE-induced HBECs. (**C**) Mitochondrial ROS levels were detected by flow cytometry (DCFH-DA). (**D**) The content of ATP in CSE-induced HBECs was detected with kits. (**E**) Western blotting was used to detect the expression of mitochondrial autophagy-related proteins such as PINK1 and Parkin. β-actin was used as an invariant internal control for calculating protein-fold changes. N=6, ** *P*<0.01.

To further explore whether puerarin prevents mitochondrial autophagy by inhibiting the dephosphorylation of FUNDC1, PH0321, a protein phosphatase inhibitor, was used to inhibit the dephosphorylation of FUNDC1. Western blotting results showed that the expression of p-FUNDC1 was decreased and mitochondrial autophagy was promoted after PH0321 treatment. The expression of p-FUNDC1 was significantly increased after intervention with high concentration of puerarin, which inhibited mitochondrial autophagy (Figure 4A). Next, the results of flow cytometry suggested that PH0321 treatment alone significantly decreased MMP level, and MMP level significantly increased after high-concentration puerarin intervention (Figure 4B). In addition, the content of ROS in cells was significantly increased after PH0321 treatment, but this trend was reversed by high concentration puerarin intervention, which significantly decreased the content of ROS in cells (Figure 4C). Furthermore, the inhibitory effect of PH0321 on ATP synthesis was reversed after high-concentration puerarin intervention (Figure 4D). Western blotting results showed that the expression of mitochondrial autophagy-related proteins such as PINK1 and Parkin was significantly upregulated after PH0321 treatment, and the expression of these two proteins was significantly downregulated after high-concentration puerarin intervention (Figure 4E). The above results indicate that puerarin prevents mitochondrial autophagy by inhibiting the dephosphorylation of FUNDC1.

### Puerarin inhibits apoptosis of CSE-induced HBECs by inhibiting mitochondrial autophagy

**Figure 5:**
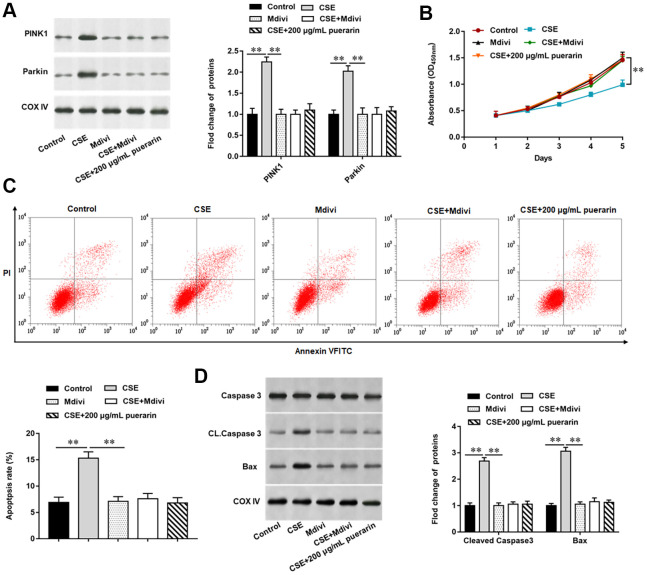
**Puerarin inhibits apoptosis of CSE-induced HBECs by inhibiting mitochondrial autophagy.** Mdivi, a mitochondrial autophagy inhibitor, treated 20% CSE-induced HBECs and then co-cultured with puerarin at a concentration of 200 μg/mL. (**A**) Western blotting was used to detect the expression of mitochondrial autophagy-related proteins such as PINK1 and Parkin. (**B**) MTT assay was used to measure the viability of CSE-induced HBECs. (**C**) Apoptosis of CSE-induced HBECs were detected by flow cytometry. (**D**) The protein expression of Cleaved caspase3 and Bax in CSE-induced HBECs were analyzed by Western blotting. β-actin was used as the loading control. N=6, ** *P*<0.01.

To further explore the effect of puerarin on apoptosis after inhibiting mitochondrial autophagy, we blocked the process of autophagy by using Mdivi, a mitochondrial division protein, as an inhibitor of mitochondrial autophagy. The results of Western blotting suggested that the expression of related proteins, such as PINK1 and Parkin, was significantly decreased after the mitochondrial autophagy process was blocked, and the changes of both proteins were consistent with the high concentration puerarin intervention (Figure 5A). Furthermore, MTT assay was used to detect cell viability and the results showed that the inhibition of 20% CSE on cell viability was reversed after treatment with mitochondrial autophagy inhibitors, which showed that the cell viability was the same as that in the high concentration puerarin intervention group (Figure 5B). Next, autophagy inhibitor treatment significantly reduced the apoptosis rate, and the expression of apoptosis- related proteins such as Cleaved caspase3 and Bax was also significantly down-regulated, which showed the same trend as the high-concentration puerarin intervention (Figure 5C, 5D).

### Puerarin inhibits mitochondrial autophagy and apoptosis in CSE-induced HBECs by activating the PI3K/AKT/mTOR signaling pathway

**Figure 6:**
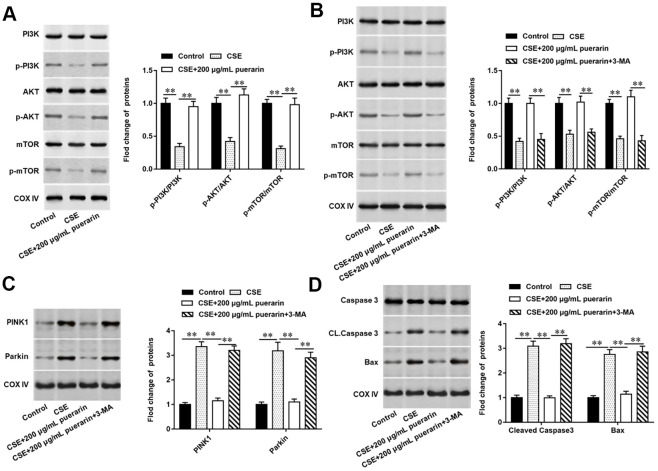
**Puerarin inhibits mitochondrial autophagy and apoptosis in CSE-induced HBECs by activating the PI3K/AKT/mTOR signaling pathway.** 3-MA, an inhibitor of PI3K, was added to 20% CES-induced HEBCs, which treated with puerarin at a concentration of 200 μg/mL. (**A**, **B**) The protein expression of total PI3K, p-PI3K, total AKT, p-AKT, total mTOR and p-mTOR in CSE-induced HBECs were analyzed by Western blotting. (**C**) Western blotting was used to detect the expression of mitochondrial autophagy-related proteins such as PINK1 and Parkin. (**D**) The protein expression of Cleaved caspase3 and Bax in CSE-induced HBECs were analyzed by Western blotting. β-actin was used as the loading control. N=6, ** *P*<0.01.

The PI3K/AKT/mTOR signaling pathway widely exists in a variety of cells and participates in a variety of physiological and pathological processes *in vivo*, and its activation has an inhibitory effect on cell autophagy. To further explore whether inhibits mitochondrial autophagy and apoptosis in CSE-induced HBECs by activating the PI3K/AKT/mTOR signaling pathway, 3-MA, a PI3K inhibitor, was used to block the pathway. It was found that the protein expression of p-PI3K, p-AKT and p-mTOR were significantly reduced in CSE-induced HBECs, while its expression was significantly reversed after high concentration puerarin intervention, suggesting that puerarin can activate the PI3K/AKT/mTOR signaling pathway (Figure 6A). Next, we blocked PI3K with 3-MA, and as expected, the expression of p-PI3K, p-AKT, and p-mTOR in cells was significantly decreased after pathway blockade compared with the high-concentration puerarin-treated group, and this signaling pathway was inhibited (Figure 6B). Western blotting results showed that the expression of mitochondrial autophagy-related proteins such as PINK1 and Parkin increased significantly after the pathway was blocked (Figure 6C), and the expression of apoptosis-related proteins such as Cleaved Casepase3 and Bax was also significantly upregulated (Figure 6D). The above results further verified that the inhibition of puerarin on mitochondrial autophagy and apoptosis in CSE-induced HBECs was accomplished by activating the PI3K/AKT/mTOR signaling pathway.

## DISCUSSION

Mitochondria are energy factories in eukaryotic cells, which can provide energy for various physiological activities of the body [7]. With the occurrence of mitochondrial oxidative phosphorylation, a lot of ROS is to be produced, and excessive ROS will lead to mitochondrial damage, causing apoptosis [19]. Therefore, clearance of damaged mitochondria through autophagy, a degradation pathway, is essential to maintain the stability of the intracellular environment.

FUNDC1 plays a key regulatory role in the biological process of mitochondrial autophagy. FUNDC1 is localized on the outer mitochondrial membrane and participates in mitochondrial autophagy caused by hypoxia and reduced mitochondrial membrane potential, in which the phosphorylated form of FUNDC1 inhibits autophagy while dephosphorylation promotes autophagy [20, 21]. Wang et al. found that mitochondrial DNA copy number and mitochondrial protein expression were significantly decreased in Ginkgolic acids-treated mouse bone marrow stromal cells, but FUNDC1 gene knockout restored Ginkgolic acids-induced changes in mitochondrial mass loss and mitochondrial membrane potential loss [22]. Another study showed that FUNDC1 plays an important regulatory role in hypoxia-induced PC12 neuroautophagy and apoptosis, and overexpression of FUNDC1 can promote autophagy and then prevent neuronal apoptosis [20]. In myocardial ischemia-reperfusion injury model, FUNDC1-mediated mitochondrial autophagy was inhibited, damaged mitochondria could not be cleared in time and accumulated in a large amount in cells, leading to microvascular endothelial cell apoptosis and barrier dysfunction [23]. In addition, some studies have found that hydrogen peroxide can up-regulate the expression of FUNDC1 by activating the ERK1/2 signaling, triggering mitochondrial autophagy, making laryngeal cancer cells more suitable for survival [24].

In recent years, more and more studies have found that mitochondrial autophagy plays an important regulatory role in the pathogenesis of COPD. Among them, Araya Jun et al. found that CS-exposed mouse airway epithelial cells showed impaired mitochondrial volume accumulation, increased oxidative modification and accelerated cell senescence, and experimental animals showed enhanced airway wall thickening and emphysema changes [25]. The upstream regulatory mechanism of mitochondrial autophagy is still unclear, and it is generally accepted that mitochondrial division is one of the important upstream regulatory factors of mitochondrial autophagy. Among them, DRP1 is a key executor. Normally, mitochondria maintain cell energy supply by controlling their number through division, but excessive mitochondrial division leads to the production of a large number of dysfunctional mitochondrial fragments, which in turn triggers mitochondrial autophagy [26–28]. In this study, we found that CSE-induced HEBCs could significantly upregulate the expression of DRP1 and FUNDC1, as well as the expression of autophagy-related proteins and apoptosis-related proteins, indicating that mitochondrial autophagy plays a positive regulatory role on apoptosis.

At present, there are few studies on the regulatory relationship between puerarin and DRP1 or FUNDC1, but the effect of flavonoids on mitochondrial autophagy through regulating DRP1 has been widely reported. Isoliglycyrrhizin isolated from Licorice attenuates neuronal cell death mediated by loss of mitochondrial membrane potential by promoting dephosphorylation of DRP1, inhibiting ROS generation and calcium ion levels in cells, and preventing glutamate-induced mitochondrial fission [29]. In a mouse model of Alzheimer's disease, icariin treatment led to a decrease in DRP1, promoted mitochondrial transport, and protected mitochondria from fragmentation, suggesting that it may be a potential therapeutic supplement for neurodegenerative diseases associated with Alzheimer's disease and other mitochondrial dysfunctions [30]. Other studies have reported that kaempferol protects neurons from succinate-mediated ischemic injury by inhibiting mitochondrial division by activating the AKT axis [31].

We found that different concentrations of puerarin could significantly reverse the damage of CES on HEBCs, which was manifested by restoring MMP level, reducing ROS generation, promoting ATP synthesis, reducing mitochondrial autophagy, and inhibiting apoptosis. We further found that puerarin inhibited mitochondrial autophagy and mitigated cell injury by inhibiting the expression of DRP1 and FUNDC1, and this protective effect may be closely related to the activation of PI3K/AKT/mTOR signaling pathway. Puerarin has been reported to alleviate oxidative stress by inhibiting lead-induced renal cell apoptosis, restoring the balance between Bax and Bcl-2, and reducing mitochondrial Cyt C release through activating the PI3K/AKT/eNOS pathway [32]. Puerarin can attenuate mitochondrial ultrastructure destruction, change MMP level to reverse cadmium-induced ATP depletion, and increase Bcl-2/Bax ratio, blocking Casepase3 activation [33]. A recent study claimed that the tuber extract of *Alisma orientale* alleviated airway inflammation and emphysema phenotype in mice by activating mTOR and inhibiting autophagy [34].

In conclusion, our study shows that puerarin can inhibit FUNDC1-mediated mitochondrial autophagy and bronchial epithelial cell apoptosis by activating the PI3K/AKT/mTOR signaling pathway to achieve cytoprotective effects, which provides a new idea for the treatment of COPD.

## MATERIALS AND METHODS

### Cell culture

The HBECs were purchased from the American Type Culture Collection (ATCC; https://www.atcc.org/) and stored in Dulbecco's modified Eagle's medium (DMEM, Sigma-Aldrich Chemical Company, St Louis, MO, USA) containing 10% FBS (Sigma-Aldrich Chemical Company, St Louis, MO, USA) and penicillin-streptomycin solution (100×; Thermo Fisher Scientific Inc., Waltham, MA, USA). Puerarin was provided by Shanghai McLean Biochemical Technology Company, batch number: C11030370, purity>98%. The suspension was prepared with sterilized saline at the concentration of 50 μg/mL, 100 μg/mL and 200 μg/mL and stored in a refrigerator at 4° C in the dark for subsequent experiments.

### Preparation of 100% cigarette smoke extract

The cigarettes used in this experiment were all Nanjing brand cigarettes. The cigarette collection system was assembled according to the cigarette support tube, gas collection bottle, buffer bottle and peristaltic pump. During gas collection, after adding about 10 mL DMEM culture medium into the gas collection bottle, start the peristaltic pump, adjust the speed about 160 r/min, and the gas flow rate is about 600 mL/min, ignite 1.5 cigarettes in turn, and shake the gas collection bottle continuously to dissolve the cigarette smoke as soon as possible. After the cigarette is burned out, the liquid obtained is recorded as 100% CSE, placed on the ultra-clean table for filtration and sterilization. After that, the CSE solution was added into DMEM to prepare the CSE mixture, which held a concentration of 20% and used as soon as possible within 30 min.

### RNA extraction and RT-qPCR

Total RNA was isolated from HEBCs by using the TRIzol (Invitrogen, Carlsbad, CA, USA). Single-stranded cDNA was synthesized with the PrimeScript Reagent Kit (Promega, USA). Real-time PCR was conducted by using SYBR Premix Ex TaqTM Kit (Applied Biosystems, Foster City, CA, USA). The reaction was run in ABI7500 Real-time PCR system (Applied Biosystems, Carlsbad, CA, USA). GAPDH was used as an endogenous control. Briefly, 2 μL of cDNA was added to 10 μL of the 1×SYBR green PCR master mix with 0.4 μL of Taq polymerase enzyme (RiboBio, China), 0.8 μL of each primer and 6 μL ddH_2_O to a final volume of 20 μL. The RT-qPCR cycling conditions consisted of: 95° C for 3 min; then 35 cycle amplification for 20 s at 95° C, 30 s at 55° C, 15 s at 72° C; followed by 1min at 72° C. The primers used in this study were synthesized from Sangon Biotech (Shanghai, China). The levels of mRNA were normalized by using the 2^-ΔΔCt^ method.

### Western blot analysis

The cells were lysed for 20 min on ice in ice-cold lysis buffer (Roche). The lysates were centrifuged at 12,000×g for 20 min at 4° C to obtain a clear lysate. The protein content of each sample was determined by using the BCA Protein Assay Kit (Thermo Scientific). Then, equal amounts of proteins (15 μg/lane) were separated on a 12% sodium dodecyl sulfate polyacrylamide gel electrophoresis (SDS-PAGE) and transferred to polyvinylidenedifluoride (PVDF) membranes (Bio-Rad, Hercules, CA, USA). The membranes were blocked in 5% (w/v) nonfat dry milk in TBST (Tris-buffered saline-0.1%Tween) at 25° C for 3 h and then incubated with the following primary antibodies: rabbit monoclonal anti-β-actin antibody (1:900, Abcam, ab179467), rabbit monoclonal anti-PINK1 antibody (1:1000, Abcam, ab216144), mouse monoclonal anti-parkin antibody (1:1500, Abcam, ab77924), rabbit polyclonal anti-Cleaved casepase3 antibody (1:1100, Abcam, ab2302), rabbit monoclonal anti-Bax antibody (1:2000, Abcam, ab32503), rabbit monoclonal anti-DRP1 antibody (1:1000, Abcam, ab184247), rabbit polyclonal anti-FUNDC1 antibody (1:500, Abcam, ab224722), rabbit monoclonal anti-PI3K antibody (1:1000, Abcam, ab32089), rabbit polyclonal anti-AKT antibody (1:1500, Abcam, ab179463), rabbit polyclonal anti-mTOR antibody (1:2000, Abcam, ab2732). The bands were incubated with horseradish peroxidase (HRP) conjugated goat anti-rabbit IgG (1:15000, Boster) for 1 h at room temperature. The bands were visualized by using an ECL Plus Chemiluminescence Reagent Kit (Pierce, Rockford, IL, USA) and were photographed by a chemiluminescence imaging system. Image J software was used to quantify the band densities.

### Detection of MMP level by flow cytometry

Cells were collected by 0.25% trypsin (containing EDTA) digestion method; 200 mesh screen filtration, cell counting, 1.5×10^5^ cells were collected; cells were resuspended by adding l mL JC-1 working solution (l×) under dark conditions, incubated at 37° C for 20 min in dark, shaken once at 2~3 min intervals; and centrifuged at 4° C for 1500 rmp; the hearts were washed twice with JC-1 staining buffer (l×) for 5 min, and the cells were resuspended with 300 μL JC-1 staining buffer (l×) for flow cytometry detection.

### Detection of ROS content by flow cytometry

Cells were collected by 0.25% trypsin (containing EDTA) digestion method; cells were filtered through 200 mesh screen, counted, and 1.5×10^5^ cells were collected; preparation of DCFH-DA working solution: incomplete cell culture solution is 1:1000; cells were resuspended by adding DCFH-DA working solution under dark conditions, and incubated at 37 degrees in the dark for 20 min; cells were washed three times with incomplete cell culture medium and resuspended with 300 μL of incomplete cell culture medium for flow cytometry detection.

### ATP content detection

Cells were collected by digestion with 0.25% trypsin (including EDTA); ATP lysate was added, mixed with shaking, ice bath for 10 min, 4° C freezing centrifuge for 12000×g Centrifuge for 5 min, extract the supernatant; prepare ATP detection working solution with ATP detection reagent: ATP detection diluent=1:4 in dark condition; 100 μL working fluid was added into 96-well plate, stand at room temperature for 2~5 minutes, then add standard diluent and sample 20 μL, and quickly mixed with a pipette gun; RUL was measured on a microplate reader and ATP concentration was calculated; protein concentration was measured at the same time, and the ratio of ATP concentration: protein concentration was shown as the final result (nmoL/mg).

### Statistical analysis

All statistical analyses were performed by using the SPSS software (ver. 22.0; SPSS, Chicago, IL, USA). The quantitative data derived from three independent experiments are expressed as mean ± SEM. Significance was determined by one-way ANOVA or *t*-test. Values of *P*<0.05 were considered statistically significant.
